# Improvements for Tissue-Chopping-Based Immunofluorescence Staining Method of Chloroplast Proteins

**DOI:** 10.3390/plants12040841

**Published:** 2023-02-13

**Authors:** Lulu Wang, Yajuan Chen, Di Niu, Mingdong Tang, Jinjie An, Shanshan Xue, Xiaomin Liu, Hongbo Gao

**Affiliations:** 1 National Engineering Research Center of Tree Breeding and Ecological Restoration, Beijing Forestry University, Beijing 100083, China; 2 College of Biological Sciences and Technology, Beijing Forestry University, Beijing 100083, China; 3 Beijing Key Laboratory of Agricultural Genetic Resources and Biotechnology, Institute of Biotechnology, Beijing Academy of Agriculture and Forestry Sciences, Beijing 100097, China

**Keywords:** tissue chopping, immunofluorescence staining, chloroplast protein, low temperature

## Abstract

Immunofluorescence staining is a very common method for the subcellular localization study of proteins. A tissue-chopping-based immunofluorescence staining method for chloroplast proteins overcomes the restriction of plant cell wall, makes the operation simpler, and uses less experimental materials. Here we provide some improvements for this method. We found that the stained tissues can be directly observed with a confocal microscope without tissue lysis. Samples maintained at a low temperature (0–4 °C) throughout the process can reduce the intensity of chlorophyll autofluorescence and the background signal. A low temperature is also good for the storage of the sample. Fluorescence signal of the stained samples can be kept for several weeks if they are stored at −20 °C. FtsZ is an essential component of the chloroplast division apparatus. We demonstrated this method with the immunofluorescence staining of FtsZ1 in wildtype Arabidopsis and some chloroplast division mutants. We also successfully tested this method by the immunofluorescence staining of FtsZ1 in many other plants, including woody plants. With these procedures, the performance of tissue-chopping-based immunofluorescence staining method are further improved.

## 1. Introduction

Chloroplasts are unique double-membrane organelles of plants. They proliferate through binary division. Chloroplast division ensures the dynamic balance of chloroplast number in plant cells. During chloroplast division, chloroplast division proteins form a ring-like super complex, which is localized to the middle of the chloroplasts [1,2]. Then the division complex constricts, separating a chloroplast into two [3]. FtsZ, which is an essential chloroplast division protein, is conserved in almost all bacteria and plants [1,4]. In plants, FtsZ has two family members, FtsZ1 and FtsZ2. FtsZ1 and FtsZ2 are localized in the chloroplast stroma, and they polymerize to form a Z-ring, which initiates chloroplast division and recruits other chloroplast division proteins [4,5]. Malfunction of a chloroplast division protein leads to abnormal FtsZ rings and blocks chloroplast division [6,7].

Immunofluorescence staining is a frequently used method in the field of cell biology. Immunofluorescence staining is often used to observe the localization of chloroplast division proteins [8,9,10,11,12]. For plants that are difficult to obtain transgenic materials for or wild samples collected in natural habitats, immunofluorescence staining is the best choice to study the localization of proteins. Moreover, the method of fluorescent labeling of proteins in transgenic plants needs fluorescent tags, which may change the structure and function of the target protein, leading to a non-native state and mis-localization of the protein in the cell [13], while immunofluorescence staining can reflect the true location of a protein in the cell.

Due to the restriction of the plant cell wall, it is difficult for the antibodies to enter the cell. Although FtsZ1 was observed to be located in the middle of chloroplasts in the wild type of *Arabidopsis thaliana* by previous sectioning-based immunofluorescence staining method [8]. Nevertheless, the immunofluorescence staining of paraffin-embedded tissue sections takes several days, and the operation is cumbersome [14].

The protoplast-based method overcomes the restriction of plant cell wall on immunofluorescence staining. With this method, leaf tissue was subjected to enzymatic digestion first, and then the immunofluorescence staining was performed with protoplasts [9]. This method has been widely used in the localization study of chloroplast division proteins [10,11]. Compared with the sectioning-based immunofluorescence staining method, enzymatic digestion is much simpler and less time-consuming, and the results can be obtained in only one day. However, this method requires preparation of poly-L-lysine slides in advance. In addition, the protoplasts are easily broken. The major drawback of this method is that protoplasts are difficult to obtain in many plants, especially woody plants.

The tissue-chopping-based immunofluorescence staining method directly chops the leaf tissue into irregular small pieces with many broken cells for the immunofluorescence staining. It does not require to embed the tissue in paraffin and perform the sectioning, as well as enzymatic digestion to generate protoplasts and the making of poly-L-lysine slides. Furthermore, it can be widely used in various plants [12].

After the development of the tissue-chopping-based immunofluorescence staining method, we made further improvements. At first, the step of tissue lysis can be omitted if the samples are observed with a confocal microscope. Secondly, operation at low temperature (0–4 °C) can greatly reduce the chlorophyll auto-fluorescence and the non-specific noise of the sample, so the signal is clearer. We also found that the storage condition of −20 °C greatly prolonged the lifetime of the sample. With these improvements, we can quickly obtain a clear view of the localization of FtsZ1 not only in Arabidopsis, but also in many woody plants, and the samples can be stored for a much longer time.

## 2. Results

### 2.1. Direct Observation with a Confocal Microscope Can Shorten the Experimental Time

**Figure 1:**
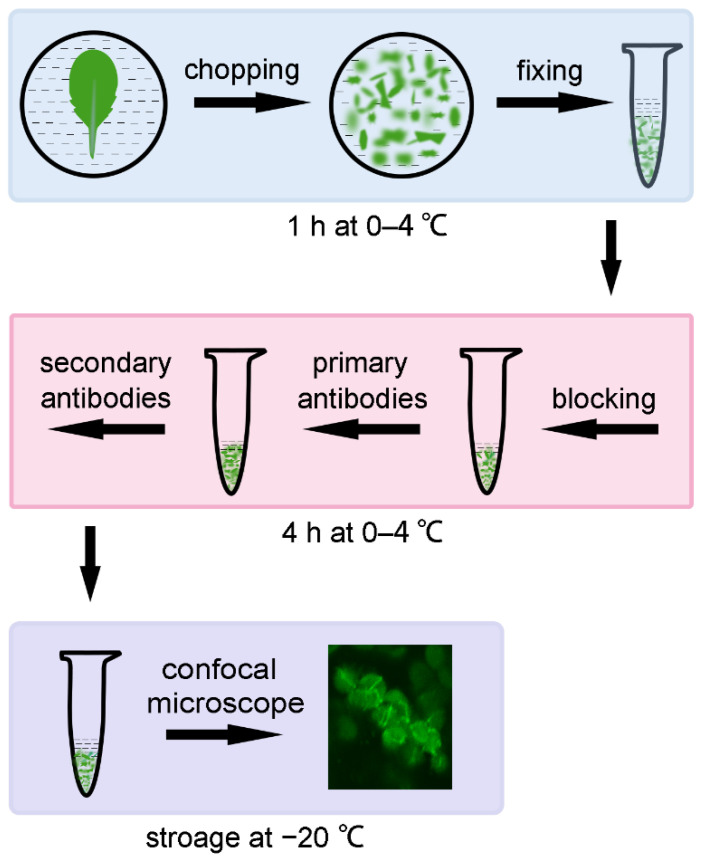
A diagram of the improvements of tissue-chopping-based immunofluorescence staining method. All the steps were operated at low temperature. Leaves were immersed in pre-cooled fixing solution and broken by a serrated blade, then transferred into a 1.5 mL tube and kept in the dark for 1 h for the fixing. After blocking for 30 min, leaf tissues were incubated with pre-cooled anti-FtsZ1 antibodies and then FITC-labeled secondary antibodies. The samples can be stored at −20 °C for several weeks. Images can be directly observed with a confocal microscope.

**Figure 2:**
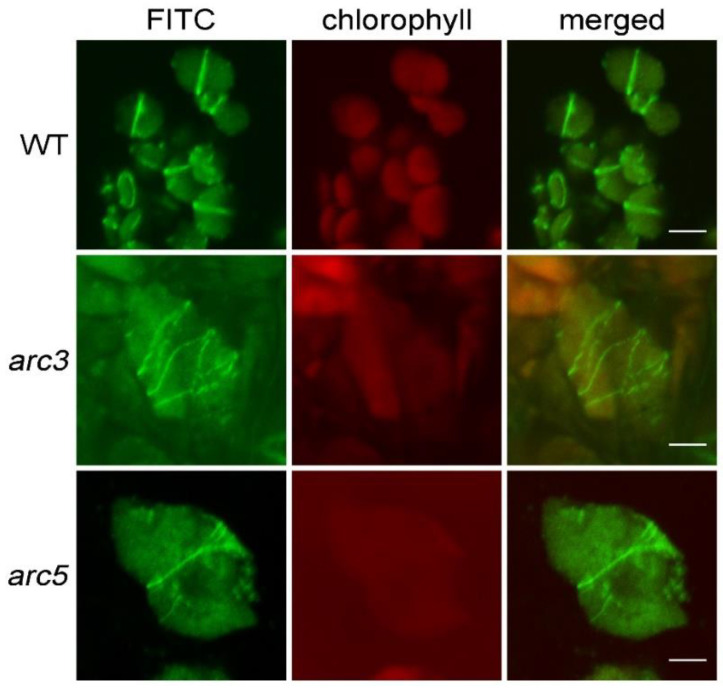
Direct observation of the immunofluorescence of FtsZ1 in Arabidopsis with a confocal microscope. WT, wild type; *arc3* and *arc5* are two chloroplast division mutants. Bars = 5 μm.

When using the tissue-chopping-based immunofluorescence staining method, we found that most of the signals existed at the edge of the broken tissue, and the signals from different cell layers interfered with each other, so sometimes it was not easy to obtain a clear view of the signal. EDTA•Na_2_ and heating treatment was used to treat the chopped leaves to make the cells disperse, but it took more time to finish the experiment [12]. So, we came up with an idea to directly observe the samples of chopped leaves, with a confocal microscope, which does not require the step of tissue lysis (Figure 1). We used the Arabidopsis leaves as the experimental material at first to study the localization of the FtsZ1 protein. The samples of chopped leaves, which were small pieces with a diameter of 2–3 mm, were directly observed with a confocal microscope after being incubated with the fluorescent secondary antibodies. FtsZ1 was found to be localized to a ring in the middle of chloroplasts in the wild type (Figure 2); while in the chloroplast division mutants *arc3* and *arc5*, multiple FtsZ1 rings were observed in the chloroplast (Figure 2). These results are consistent with the previous reports [7,15], indicating this arrangement is feasible. The omission of the tissue lysis not only reduces the steps of operation, shortens the total experimental time, but also makes it easier to obtain the signal of the target protein.

### 2.2. Low Temperature Can Reduce Chlorophyll Autofluorescence and the Background Noise Signal

**Figure 3:**
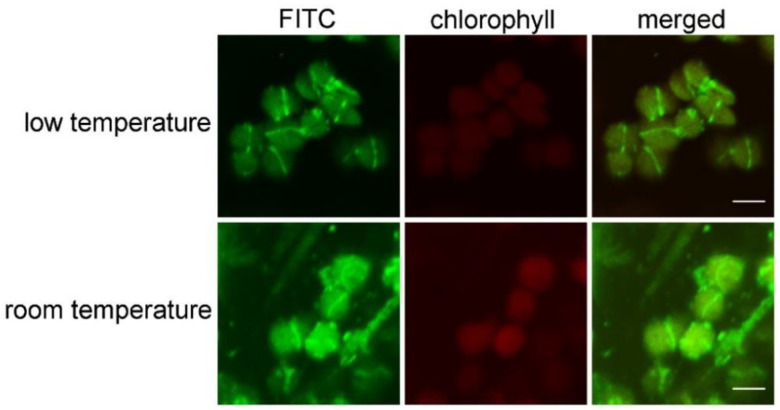
A comparison of the fluorescence signal between samples prepared at low temperature (0–4 °C) and samples prepared at room temperature. Images from two samples were captured with the same exposure condition. Bars = 5 μm.

In order to make the fluorescence signal of the target proteins clearer and reduce the background signal, we tested the effect of low temperature. Two leaves with similar growth states from the same wild-type Arabidopsis plant were taken. The experiments with these two leaves were carried out in parallel. One was at room temperature, and the other was at low temperature (0–4 °C), respectively. After the immunofluorescence staining, these two samples were observed with the same exposure condition. The results showed that the chlorophyll autofluorescence of the samples prepared at low temperature was significantly weaker than those prepared at room temperature. The decrease in chlorophyll autofluorescence provided a striking contrast with the fluorescence of the target protein, making the signal of target proteins clearer (Figure 3). Meanwhile, background noise signals of the samples prepared at low temperature were reduced to a certain extent. Thus, a better result can be obtained at low temperature (0–4 °C) than the room temperature.

### 2.3. Storage at −20 °C Can Effectively Prolong the Lifetime of the Sample

**Figure 4:**
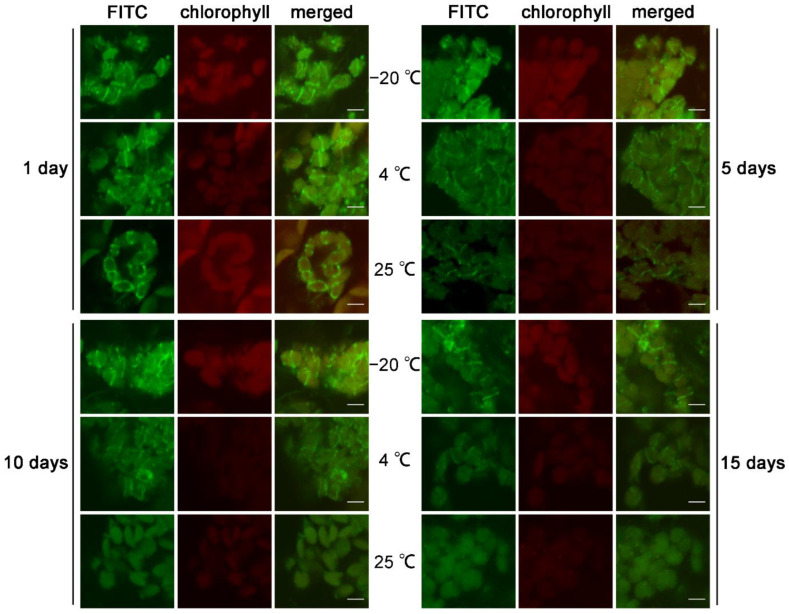
Storing at −20 °C prolongs the lifetime of the samples. The stained protein was FtsZ1. A comparison of the signal intensity for samples stored at −20 °C, 4 °C, and 25 °C on the 1st day, 5th day, 10th day, and 15th day of the storage. Images from these samples were captured with the same exposure condition. Bars = 5 μm.

Next, we explored the lifetime of samples under different storage temperature conditions. After the immunofluorescence staining of FtsZ1, the samples from the wild type were evenly divided into three parts. We stored these three parts of the same sample at room temperature, 4 °C, and −20 °C, respectively, observed these stored samples on the 1st, 5th, 10th, and 15th day, and compared the intensity and quantity of the signals of samples under different storage conditions (Figure 4). The results indicated that on the first day, there was no obvious difference among the signals from samples at different temperature conditions. On the 5th day, the signal intensity from samples stored at room temperature decreased obviously. The signal intensity of samples stored at 4 °C also decreased obviously. However, there was no obvious change in the signal for samples stored at −20 °C. On the 10th day, the signal intensity of samples at room temperature was weak. The signal intensity of the samples at 4 °C was further reduced. The signal intensity of the samples stored at −20 °C was only slightly reduced. On the 15th day, the signal completely disappeared in the samples stored at room temperature. The signal intensity of samples stored at 4 °C was very weak, and the number of chloroplasts with signals sharply decreased. The signal intensity of samples stored at −20 °C for 15 days was obviously reduced but good for observation. In summary, our results showed that a storage condition of −20 °C can prolong the lifetime of the sample.

### 2.4. The Improved Immunofluorescence Staining Method Can Be Widely Used in Other Species

**Figure 5:**
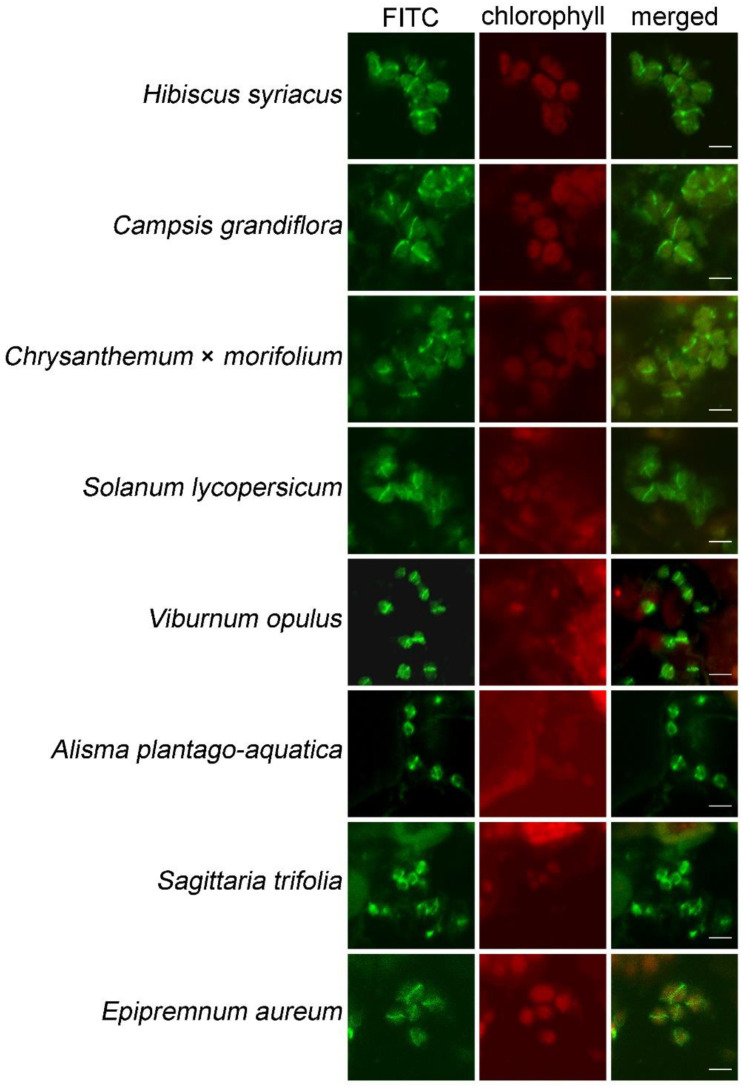
Localization study of FtsZ1 in various plant species with this improved immunofluorescence staining method. Plant species shown are *Hibiscus syriacus* L., *Campsis grandiflora* (Thunb.) Schum., *Chrysanthemum* × *morifolium*, *Solanum lycopersicum* L., *Viola philippica* Cav., *Alisma plantago-aquatica* L., *Sagittaria trifolia* subsp. *leucopetala* (Miquel) Q. F. Wang ‘Florepleno’, *Epipremnum aureum* (Linden et Andre) Bunting. Bars = 5 μm.

To further test the effect of these improvements in other plants, we used this method to analyze the localization of FtsZ1 protein in 56 plant species. The signal of FtsZ1 can be observed in 35 species (Table 1). In these plants, FtsZ1 is localized to the middle of chloroplasts and forms a ring (Figure 5), which is similar to the localization of FtsZ1 in *Arabidopsis* (Figure 2). The signal of FtsZ1 cannot be observed in the other 21 species (Supplemental Table S1). Overall, this improved method is also applicable to many other plant species.

## 3. Discussion

Immunofluorescence staining is a widely used method to study the localization of proteins in cells. In this study, we improved the tissue-chopping-based immunofluorescence staining method [12]. We kept the whole process of sample preparation at low temperature (0–4 °C). After the immunofluorescence staining, the samples can be observed directly with a confocal microscope. The samples were stored at −20 °C. Although these measures are common, they are very useful.

Our improved method has a lot of advantages. After the incubation with primary antibodies and secondary antibodies, the samples can be directly observed with a confocal microscope without EDTA•Na_2_ and heating treatment. Moreover, without tissue lysis, the cells with signals are concentrated on the edge of the broken tissue, so it is easier to find the signals of the target proteins and it also avoids damages to the cells during sample preparation. Secondly, we observed 56 other plant species with this improved method, and FtsZ1 of 35 plant species was observed to localize in the middle of chloroplasts as a ring. Thirdly, we found that keeping the whole process of sample preparation at low temperature (0–4 °C) can reduce the chlorophyll autofluorescence, increase the contrast of the signal, and make the signal of the target proteins clearer. At last, we stored the prepared samples at −20 °C, which greatly prolonged the lifetime of the signal of target proteins. These procedures make the immunofluorescence staining method more convenient and save the time.

We believe that there is space for improvements of this method. When we observed the signals of the target proteins, we tried various methods, but could not eliminate the background signal in cells. If the background signal can be eliminated or significantly reduced, the signals of target proteins will be clearer and the quality of the images will be enhanced. In addition, although the localization of the target proteins can be observed in many plant species by the improved immunofluorescence method, there are some plants in which we cannot observe the signal of the target proteins by this improved method. We speculate there may be several reasons. A possible reason is the features of plant tissue. The first step of the tissue-chopping-based immunofluorescence staining method is to cut plant tissues with a saw-shaped blade. During the experiment, we found that the leaf surfaces of some plants are covered with a thick waxy cuticle and some plants have thick leathery leaves, resulting in hard plant leaves that are not easy to form irregular wounds. As a result, it is difficult for antibodies to enter the cells and few signals were observed. The second possible reason is the structure of plant cells. The components or structures of the cell walls in some plants are relatively special. For example, we cannot observe the signals of the target proteins by this improved method in almost all gramineous plants. We hope that through continuous improvements, this tissue-chopping-based immunofluorescence staining method will be better and more applicable to different plants in the future.

## 4. Material and Methods

### 4.1. Plant Materials and Growth Conditions

The *Arabidopsis thaliana* plants used in this study are Columbia-0 (Col), *arc3,* and *arc5* mutants [16,17]. Arabidopsis seeds were surface-sterilized, sown on half-strength Murashige and Skoog (MS) plates, and stratified at 4 °C for 48 h. Ten-day-old seedlings were transferred into soil after germination. Plants were grown in controlled-environment chambers with 16 h of light and 8 h of dark at 21 °C and the relative humidity is 40–60%. Leaves from 30-day-old plants were used for the experiments. Another 56 plant species (Table 1 and Supplemental Table S1), such as *Hibiscus syriacus* L., *Campsis grandiflora* (Thunb.) Schum., *Solanum lycopersicum* L. and so on, were grown on the Beijing Forestry University campus.

### 4.2. Tissue Breaking and Fixing of Leaves

Arabidopsis leaves from 30-day-old soil-grown plants or leaves from other plants were collected and immersed in fixing solution (0.4 M Mannitol, 20 mM KCl, 20 mM MES (pH 5.7), 4% paraformaldehyde) in Petri dishes, and cut into pieces with a saw-shaped blade which was taken from GLAD ClingWrap (W300N, Clorox China Limited). Then the broken tissue and the fixing solution were transferred into a 1.5 mL tube and kept in the dark for 1 h.

### 4.3. Immunofluorescence Staining

After fixing, leaf tissues were gently washed three times with 1 × PBS (137 mM NaCl, 2.7 mM KCl, 10 mM Na_2_HPO_4_•12H_2_O, 2 mM KH_2_PO_4_, pH 7.4) and each time for at least 5 min. Subsequently, the sample was covered with 1 mL blocking solution (5% BSA in 1 × PBS with 0.15% TritonX-100) and incubated at room temperature for 30 min. Next, the leaf tissues were incubated with 200 μL anti-FtsZ1 antibodies (a dilution of 1:100 with blocking solution) for 2 h at room temperature. The samples were washed three times with 1 × PBS (or 1 × PBS containing 5% BSA). Then the tissues were incubated with 200 μL Goat anti-Rabbit FITC-conjugated secondary antibodies (JX3004, Jiaxuan Biotech, Beijing) (1:100 dilution in 5% BSA) for 1 h in the dark. The samples were washed three times with 1 × PBS (or 1 × PBS containing 5% BSA). The experiments were carried out either at room temperature or at 0–4 °C.

### 4.4. Storage of Samples

The samples were immersed in 1 mL freshly prepared anti-fading buffer (5 mM Na-Ascorbate, 15 mM Na_2_HPO_4_ pH 9.0, 50% glycerin) and stored at room temperature, 4 °C and −20 °C, respectively.

### 4.5. Confocal Microscopy and Image Analysis

Samples were observed with a Nikon inverted fluorescence micro-scope TE2000-E equipped with 60 × objectives and images were captured with a Nikon D-Eclipse A1 spectral confocal laser scanning system. The excitation wavelengths with 100% power of laser were 488 nm (FITC) and 638 nm (chlorophyll). The high voltage of 488 nm was 110 ms (FITC) and 638 nm (chlorophyll) was 60 ms. Image analysis was carried out by ImageJ (http://rsbweb.nih.gov/ij/, accessed on 15 July 2021; version 1.52V) and Photoshop (Adobe Photoshop CC 2015) for contrast/brightness adjustments and cropping.

## Figures and Tables

**Table 1:** A list of the plant species in which the signal of FtsZ1 can be observed with this improved immunofluorescence staining method.

A List of the Plant Species with Signals
*Sophora japonica* *Sophora japonica* ‘Oligophylla’ *Styphnolobium japonicum* ‘Pendula’ *Yulania denudata* *Lonicera maackii* (Rupr.) Maxim. *Viburnum opulus* subsp. *calvescens* (Rehder) Sugimoto *Kolkwitzia amabilis* Graebn. *Jasminum nudiflorum* Lindl. *Syringa reticulata* subsp. *amurensis* (Ruprecht) P. S. Green and M. C. Chang *Aesculus chinensis* Bunge *Koelreuteria paniculata* Laxm. *Kerria japonica* (L.) DC. *Amygdalus persica* ‘Atropurpurea’ *Prunus triloba* ‘Multiplex’ *Diospyros lotus* *Sambucus williamsii* Hance *Hibiscus syriacus* L. *Campsis grandiflora* (Thunb.) Schum. *Parthenocissus quinquefolia* *Solanum lycopersicum* L. *Scadoxus pole-evansii* (Oberm.) Friis and Nordal *Chenopodium album* L. *Taraxacum mongolicum* Hand.-Mazz. *Erigeron canadensis* L. *Inula japonica* Thunb. *Lactuca indica* L. *Chrysanthemum* × *morifolium* *Lythrum salicaria* L. *Viola philippica* Cav. *Orychophragmus violaceus* (Linnaeus) O. E. Schulz *Hosta plantaginea* *Sagittaria trifolia* subsp. *leucopetala* (Miquel) Q. F. Wang *‘Florepleno’* *Alisma plantago-aquatica* L. *Epipremnum aureum* (Linden et Andre) Bunting *Lemna minor* L.

## Data Availability

Not applicable.

## Supplementary Materials

The following supporting information can be downloaded at: https://www.mdpi.com/article/10.3390/plants12040841/s1, Supplemental Table S1. A list of the species in which the signal of FtsZ1 cannot be observed with this improved immunofluorescence staining method.
