# Supplementary material for: Improvements for Tissue-Chopping-Based Immunofluorescence Staining Method of Chloroplast Proteins
Source: Plants (Basel). 2023 Feb 13;12(4):841. doi: 10.3390/plants12040841 (PMC9963192; doi:10.3390/plants12040841)
Supplement: Supplementary file 1 [file plants-12-00841-s001.zip › plants-1857554-supplementary.pdf]

Table S1. A list of the species in which the signal of FtsZ1 cannot be observed with this improved immunofluorescence staining method.

| A list of partial species in which FtsZ1 cannot be observed |
|---|
| *Sorbaria sorbifolia* (L.) A. Br. |
| *Cotoneaster horizontalis* Dcne. |
| *Prunus tomentosa* (Thunb.) Wall. |
| *Malus × micromalus* Makino |
| *Spiraea × vanhouttei* (Briot) Carriere |
| *Potentilla freyniana* Bornm. |
| *Fraxinus chinensis* Roxb. |
| *Dyringa vulgaris* |
| *Forsythia suspensa* (Thunb.) Vahl |
| *Platanus occidentalis* |
| *Cornus officinalis* Siebold and Zucc. |
| *Eucommia ulmoides* Oliver |
| *Weigela florida* (Bunge) A. DC. |
| *Liriodendron chinense × tulipifera* |
| *Acer truncatum* Bunge |
| *Quercus mongolica* Fischer ex Ledebour |
| Parthenocissus tricuspidata |
| *Arundo donax 'Versicolor'* |
| *Bambusa basihirsuta* McClure |
| *Schoenoplectus tabernaemontani* (C. C. Gmelin) Palla |
| *Hemerocallis fulva* (L.) L. |
